# Is redox signaling a feasible target for overcoming multidrug resistance in cancer chemotherapy?

**DOI:** 10.3389/fphar.2014.00286

**Published:** 2014-12-23

**Authors:** Manuela Polimeni, Elena Gazzano

**Affiliations:** Department of Oncology, University of TurinTurin, Italy

**Keywords:** MDR, oxidative stress, cancer, antioxidants, chemoterapy

Under physiological conditions, a balance between oxidants and antioxidants exists. Reactive oxygen species (ROS), are continuously generated by aerobic cells and eliminated through scavenging systems to maintain redox homeostasis. The two main sources of ROS are mitochondria and the NADPH oxidases family, but ROS are produced also by the cytochrome P450 system, xanthine oxidase and nitric oxide synthase (Holmstrom and Finkel, 2014). Because of ROS reactivity toward lipids, proteins and DNA, spatial and temporal regulatory strategies exist to regulate their intracellular levels. Excessive ROS levels are controlled by specific intracellular enzymes, such as superoxide dismutase (SOD), glutathione peroxidase, catalase, thioredoxin reductase, and glutathione S-transferase (Glasauer and Chandel, 2014).

Cells aim to maintain a redox homeostasis: low levels of ROS, which are locally produced, can be potent mitogens and are required for various biological processes such as cell survival, growth and proliferation, angiogenesis, gene expression (Finkel, 2012). In contrast, changes in redox balance result in oxidative stress and aberrant cell signaling. Many studies have shown the critical role of detoxifying enzymes and antioxidant proteins in modulating the correct balance between apoptosis and carcinogenesis. Firstly, higher ROS levels could play a causal role in cancer development and progression by inducing DNA mutations, genomic instability, aberrant pro-tumorigenic signaling. After that, cancer cells adapt to oxidative stress and counteract the potential toxic effects of ROS to promote cell proliferation, survival and metabolic adaptation to the tumor microenvironment: sustained cell proliferation and mitogenic signaling (Weinberg and Chandel, 2009), increased cell survival and disruption of cell death signaling (Clerkin et al., 2008), epithelial to mesenchymal transition, metastasis (Nishikawa, 2008) and angiogenesis (Ushio-Fukai and Nakamura, 2008). Therefore, cancer cells are dependent on maintaining high enough ROS levels (redox imbalance) and an altered redox environment that allow for pro-tumorigenic cell signaling without inducing cell death (Glasauer and Chandel, 2014).

Despite new discoveries and some clinical successes, the major obstacle to the effective treatment of human cancer is still the development of multidrug resistance (MDR) (Simon and Schindler, 1994). The mechanisms involved are complex and multifactorial (Baird and Kaye, 2003), but it is now accepted that classical redox transcription factors (NF-kB, HIF, p53, PI3K, AP-1) are involved in the development of MDR. Both carcinogenesis and MDR are frequently associated with an increased oxidative stress and activation of redox metabolism: this could affect the efficacy of cancer treatments by multiple mechanisms, including apoptosis, angiogenesis, metastasis, inflammatory reaction, and chemosensitivity (Morrow et al., 2006; Kuo, 2009). As a result, to balance oxidative stress, cancer cells increase their antioxidant capacity: according to our experience, for example, glutathione (GSH) plays a pivotal role in MDR development.

Besides classical redox pathways many studies recently focused on other redox-sensitive factors. Nuclear factor-erythroid 2 related factor 2 (Nrf2), via its binding to antioxidant response element (ARE), regulates the expression of cytoprotective genes: classical antioxidant enzymes including SOD and catalase, phase 2 detoxifying enzymes, and stress response proteins such as heme oxygenase 1 (Kaspar et al., 2009). In quiescent conditions, Nrf2 is anchored in the cytoplasm to Kelch-like ECH-associated protein 1 (KEAP-1), an adaptor protein which facilitates the Nrf2 ubiquitination and proteasomal degradation. Nrf2 nuclear accumulation is mainly mediated by KEAP-1-dependent turnover: its thiol-modification has long been associated to a primary response to ROS production (Dinkova-Kostova et al., 2002). Owing to its cytoprotective functions, Nrf2 has been traditionally studied in the field of chemoprevention; however, its overexpression or hyperactivation may participate in tumorigenesis of a wide number of solid cancers and leukemias (Nioi and Nguyen, 2007; Shibata et al., 2008; Homma et al., 2009). Moreover, Nrf2 activity is connected with oncogenic kinase pathways, structural proteins, hormonal regulation, other transcription factors, and epigenetic enzymes involved in the pathogenesis of various tumors (Gañán-Gómez et al., 2013). In addition to protecting cells from ROS, Nrf2 seems to play a direct role in MDR acquisition in many cancer types. Recent studies suggested a dark side of Nrf2 pathway by showing that high level of Nrf2 can promote cancer formation and contribute to chemoresistance (Hayes and McMahon, 2006; Lau et al., 2008; Wang et al., 2008; Kensler and Wakabayashi, 2010; Gañán-Gómez et al., 2013). For example, a greater nuclear accumulation of Nrf2 leads to constitutive overexpression of ARE-containing genes including drug efflux pumps, which facilitate the development of resistance (Meijerman et al., 2008). The expression of Nrf2 in cancer cells is increased during acquired resistance to doxorubicin and tamoxifen in ovarian and breast cancer cells (Kim et al., 2008; Kaspar et al., 2009). In addition, stable overexpression of Nrf2 or its upregulation by tert-butylhydroquinone resulted in enhanced resistance of cancer cells to some chemotherapeutic agents (Wang et al., 2008). High expression of Nrf2 and its target genes in MCF-7 and MDA-MB-231 mammospheres compared to corresponding adherent cells is associated with increased resistance to taxol and anchorage-independent growth (Wu et al., 2014). Moreover, transport activities of several MDR-associated proteins (MRPs) are regulated by GSH availability, and γ-glutamylcysteine synthetase (GCS) is the rate-limiting enzyme for its *de novo* biosynthesis. Transcriptional regulation of γ-GCS and MRP1 expression is mediated by an ARE that contains a consensus sequence for Nrf2; so, co-regulation of γ-GCS and MRP1 would facilitate the efflux activity (Glasauer and Chandel, 2014).

APE-1/Ref-1 (Apurinic-apyrimidinic endonuclease 1/Redox Factor 1) is a multifunctional protein with both DNA repair and transcriptional regulatory activities by facilitating DNA binding of numerous transcription factors involved in cancer promotion and progression, (AP-1, NF-κ B, HIF, CREB, p53) (Luo et al., 2008). APE-1 requirement for cellular survival and its frequent overexpression in tumor cells strongly suggests a fundamental role in preventing cell death and controlling proliferation (Tell et al., 2005). Elevated APE-1 levels have been found in ovarian, cervical, prostate cancers, rhabdomyosarcoma and germ cell tumors (GCTs) correlating with the tumors radiosensitivity (Evans et al., 2000). Furthermore, immunohistochemistry in sections of GCTs from patients with testicular cancer of various histologies revealed high levels of APE-1 expression, suggesting a relation with their relative resistance to therapy (Robertson et al., 2001). Other evidences revealed that APE-1 contributes to alkylating agent resistance (Silber et al., 2002) or radioresistance in human glioma cells (Naidu et al., 2010), promotes resistance to radiation plus chemotherapy in medulloblastoma and primitive neuroectodermal tumors and in pediatric ependymomas (Bobola et al., 2011). Moreover, APE-1, preferably in the acetylated form, stably interacts with Y-box-binding protein 1 and enhances its binding to the Y-box element, leading to the activation of the *MDR1* gene. Indeed, a systematic increase in both APE-1 and MDR1 expression was observed in non-small-cell lung cancer tissue samples (Chattopadhyay et al., 2008).

Forkhead box O (FoxO) proteins are a family of transcription factors that, regulated by several stimuli, modulate genes involved in differentiation, proliferation, survival, apoptosis, migration and DNA repair (Dansen and Burgering, 2008; Yang and Hung, 2009). Upon exposure to an oxidative stress, FoxOs can lead to apoptosis or adaptive responses, depending on the entity of the stress. FoxO proteins have an important role in regulating cellular antioxidant defenses through the induction of genes encoding Mn-SOD and catalase; therefore, loss of FoxO function could contribute to increase the cellular ROS levels, eventually leading to DNA damage (Dansen and Burgering, 2008). FoxOs are deregulated in several tumors including breast and prostate cancers, glioblastoma, rhabdomyosarcoma, and leukemia (Myatt and Lam, 2007). During tumor development, the inhibition of FoxO3 transcriptional activity promotes cell transformation, cancer progression, and angiogenesis (Yang and Hung, 2009). Therefore, FoxOs inactivation seems to be an important step in carcinogenesis and increasing their activity could represent a therapeutic strategy (Myatt and Lam, 2007; Yang and Hung, 2009). Additionally, under continuous stress FoxOs could also induce the expression of important genes for drug efflux and antioxidant defense: the same molecules are responsible for not only the initial therapeutic response to cancer drugs, but also the subsequent acquisition of drug resistance (Zhang et al., 2011; Gomes et al., 2013). Sustained FoxO activation may promote MDR and cell survival: FoxO3 and FoxO1 induce MDR1 expression respectively in K562 leukemic cells and adriamycin-resistant breast cancer cells (Han et al., 2008; Yang and Hung, 2009). In addition, the proximal promoter region of the human *MRP2* gene contains four putative FoxO binding sites, and its transcription was stimulated by FoxO1 overexpression in MCF-7 cells (Choi et al., 2013). FoxO1 expression was distinctively upregulated in paclitaxel resistant cell line and enhanced by exposure to paclitaxel with subcellular translocation; in addition, FoxO1 overexpression was frequently observed in cancer tissue samples from chemoresistant patients (Goto and Takano, 2009). Paradoxically, cytostatic and cytotoxic effects of a diverse spectrum of anti-cancer drugs, such as paclitaxel, doxorubicin, lapatinib, gefitinib, imatinib, and cisplatin, are mediated through the FoxO3 activation and/or the inhibition of its direct target FOXM1. Moreover, there are also studies in which cisplatin-resistant cells had decreased levels of FoxO3 expression and were more sensitive to the anticancer agent mithramycin than their parental cells: FoxO3 knockdown increased cell proliferation and resistance to cisplatin (Shiota et al., 2010). However, deregulation of FoxOs has been recently found also in leukemia, where active FoxOs maintain leukemia stem cells and stimulate drug resistance genes, contributing to leukemogenesis (Zhu, 2014).

Several approaches have been undertaken to combat MDR. In the light of these findings, modulation of cellular redox levels could have important implications for the development of potential anticancer therapies. Several reports have demonstrated that Nrf2 silencing in cancer cells could decrease cell proliferation and enhance sensitivity to chemotherapeutic agents in lung, gallbladder, and ovarian tumors (Meijerman et al., 2008; Singh et al., 2008). Very recently, brusatol, an inhibitor of the Nrf2 pathway, was discovered to suppress Nrf2 level and its target genes, enhancing intracellular ROS, sensitizing MCF-7 and MDA-MB-231 mammosphere cells to taxol and reducing anchorage-independent growth (Wu et al., 2014). Reducing the APE-1 amount in cancer using RNA interference and antisense oligonucleotide technology sensitizes tumor cells to a variety of chemotherapeutic agents. For example treatment of a human pancreatic cancer cell line (Panc-1) with antisense oligonucleotides to APE-1 resulted in a dramatic increase in gemcitabine sensitivity (Lau et al., 2004). Therefore, selective APE-1 activity inhibition could have potential therapeutical significance and be a promising avenue to develop novel cancer treatments (Jiang et al., 2008; Bapat et al., 2009). APE-1 may be a useful target for modifying radiation tolerance: the inhibitors lucanthone and CRT004876 were employed, the former a thioxanthene previously under clinical evaluation as a radiosensitizer for brain tumors and the latter a more specific inhibitor (Naidu et al., 2010); knockdown of APE-1 gene expression may significantly sensitize pancreatic cancer cells to radiotherapy (Chen et al., 2013). Finally, some studies demonstrated that Bcl-2 could directly interact with APE-1 via its BH domains: gossypol, a Bcl-2 homology 3 (BH3)-mimetic agent binds to the BH3 domain of Bcl-2 family members and inhibits the repair activity and the redox function of APE-1 (Qian et al., 2014). Because of its pivotal role in drug sensitivity as well as resistance, the complex of FoxO could be a viable strategy for cancer treatment and drug resistance overcoming, while in cancer patients might also help to predict and monitor their clinical response to chemotherapy.

Although in the past antioxidants were seen as tumor suppressors, recent research uncovered the “dark side of antioxidants” (Wang et al., 2008; Sayin et al., 2014), which are used by cancer cells to promote survival and growth. The dependence of cancer cells on ROS homeostasis may represent the cancer cell's “Achilles Heel” and could be potentially exploited to target them therapeutically: pro-oxidant cancer therapy can affect the different ROS production and redox regulation between normal and cancer cells. At last, recent discoveries about Nrf2, APE-1, FoxO and their potential contribution in the development, maintenance and evolution of MDR in cancer, open a novel therapeutic window for cancer treatment. High levels of ROS can be toxic to cancer cells and potentially induce cell death via oxidative stress while sparing normal cells. Therein, redox modulators could be promising tools in MDR cancer prevention and treatment; nevertheless, because of the complexity underlying drug resistance, it will be necessary to do careful antioxidant profiling of tumor cells to identify clinically relevant therapeutic targets.

## Conflict of interest statement

The authors declare that the research was conducted in the absence of any commercial or financial relationships that could be construed as a potential conflict of interest.
